# Layer-by-Layer Investigation of Ultrastructures and Biomechanics of Human Cornea

**DOI:** 10.3390/ijms23147833

**Published:** 2022-07-15

**Authors:** Erick Rafael Dias Rates, Charles Duarte Almeida, Elaine de Paula Fiod Costa, Roberta Jansen de Mello Farias, Ralph Santos-Oliveira, Luciana Magalhães Rebelo Alencar

**Affiliations:** 1 Laboratory of Biophysics and Nanosystems, Department of Physics, Federal University of Maranhão, Campus Bacanga, São Luís 65080-805, MA, Brazil; erick.rates@discente.ufma.br (E.R.D.R.); charlesduartealmeida@gmail.com (C.D.A.); 2 Department of Medicine, Federal University of Maranhão, Praça Gonçalves Dias—Centro, São Luís 65020-070, MA, Brazil; epf.costa@ufma.br; 3 Presidente Dutra Unit, University Hospital of the Federal University of Maranhão (HUUFMA), São Luís 65020-070, MA, Brazil; roberta.jansen@gmail.com; 4 San Francisco Eye Institute, São Luís 65076-090, MA, Brazil; 5 Laboratory of Nanoradiopharmaceuticals and Radiopharmacy, Rio de Janeiro State University, Rio de Janeiro 23070-200, RJ, Brazil; presidenciaradiofarmacia@gmail.com; 6 Brazilian Nuclear Energy Commission, Nuclear Engineering Institute, Rio de Janeiro 21941-906, RJ, Brazil

**Keywords:** cornea, biophysics, ultrastructure, biomechanics, AFM

## Abstract

The cornea is an avascular, innervated, and transparent tissue composed of five layers: the epithelium, Bowman’s layer, stroma, Descemet’s membrane, and endothelium. It is located in the outermost fraction of the eyeball and is responsible for the refraction of two-thirds of light and protection from external mechanical damage. Although several studies have been done on the cornea on the macroscopic scale, there is a lack of studies on the micro-nanoscopic scale, especially an analysis evaluating the cornea layer by layer. In this study, atomic force microscopy (AFM) was employed to assess four layers that form the cornea, analyzing: adhesion, stiffness, and roughness. The results showed microvilli in the epithelial and endothelial layers, pores in the basement membrane, and collagen fibers in the Stroma. These data increase the knowledge about the human cornea layers’ ultrastructures and adds new information about its biophysical properties.

## 1. Introduction

The cornea is a complex, avascular, and transparent tissue that acts as the outer protective layer of the eye. The cornea is mainly constituted by a collagenous extracellular matrix (ECM) layer embedded in keratocytes, sustained by a layer of stratified epithelium and an innermost layer of the endothelium [1,2]. It presents typical characteristics of soft biological materials: incompressibility, non-linear elastic behavior, and viscoelasticity [3]. Histologically, the cornea is divided into five layers: the epithelium, Bowman’s layer, Stroma, Descemet’s membrane, and endothelium [4,5,6]. The corneal stroma comprises 90% of the total corneal thickness and consists of keratocytes, collagenous lamellae, and proteoglycan ground substance. The posterior-most stroma is acellular and strongly adherent to the underlying Descemet’s membrane, the remainder of the stroma (the pre-Descemet Dua’s layer), which can be exploited in deep lamellar keratoplasty [7].

Nowadays, there are several techniques, such as slit-lamp biomicroscopy (SLB), endothelial specular microscopy, confocal microscopy, optical coherence tomography, Sheimpflug imaging, and ultrasound biomicroscopy, for corneal assessment, which makes the evaluation of corneas fast, reliable, and simple, helping in the identification and management of several diseases [8]. However, most of the techniques used in corneal studies analyze the cornea at the macroscopic level, leading to the micro-nanoscopic level being unraveled and unknown. In this direction, the use of AFM to study ocular tissue is presented as a promising tool that may provide information that can correlate nanometric structures with changes in their functions at the macroscopic level [9,10,11]. For instance, Yamamoto et al. [12] showed that fibrils’ D-periodicity had different signatures when comparing corneal and scleral fibrils.

In addition to ultrastructure assessment, AFM provides information regarding individual tissues and cell mechanical properties [13], such as viscoelasticity, roughness, and stiffness [10,12,13,14,15]. Thus, to better understand the behavior of the cornea at the micro-nanoscopic level, we performed a layer-by-layer AFM screening of the human cornea.

## 2. Materials and Methods

### 2.1. Ethical

All procedures were approved by the Research Ethics Committee (CAAE No. 16031219.0.0000.5086) and the Eye Bank of the Federal University of Maranhão.

### 2.2. Sample Acquisition and Preparation

Corneal samples were obtained from the Eye Bank of the Federal University of Maranhão (HUUFMA, São Luís, Maranhão, Brazil) and preserved in Optsol GS. Corneas were cut into four quadrants, and each quadrant was used to analyze a different layer. For the analysis of the inner layers beyond the epithelium, the cornea was gently scraped with a No. 15 scalpel by the medical professional of the eye bank. Light microscopy was used to observe the samples previously to ensure the analyzed layer. Then, the tissue was fixed to the magnetic disk of the equipment with an instant adhesive based on cyanoacrylate for the AFM analysis.

### 2.3. Atomic Force Microscopy (AFM) Ultrastructural and Nanomechanical Experiments

#### 2.3.1. AFM Setup

The atomic force microscopy analysis was performed using an AFM Multimode 8 (Bruker, Santa Barbara, CA, USA) in PeakForce Quantitative NanoMechanics (QNM) mode. In this operating mode, the probe oscillated with a frequency well below the resonant frequency of the cantilever (in our 1 kHz experiment), and with each oscillation, it acquired a force curve. The information on height and nanomechanical properties, such as stiffness and adhesion, was obtained concomitantly with the acquisition of force curves. Probes with a 0.4 N/m cantilever nominal spring constant and a 2 nm tip radius (Scanasyst Air, Bruker Probes) were used in all measurements. However, the real spring constant of each cantilever was calibrated according to the thermal tune method [16]. The real tip radius was calibrated on a titanium surface (Ti Roughness Sample, Bruker) by scanning a 2 × 2 µm area on this surface, and the actual radius was obtained in Tip Qualification Control mode in the Nanoscope Analysis 2.0 software (Bruker, CA, USA).

The PeakForce Quantitative NanoMechanics (QNM) operating mode is an extension of the intermittent contact mode (tapping mode) that allows the acquisition of force curves in each pixel of an image obtained at high frequencies (0.5–2 kHz), allowing the quantitative measurement of the sample properties at the nanoscale, generating high-resolution maps (with resolutions compatible with those of topographic images). As the QMN mode controls the force applied to the sample by the tip, the indentations on the sample’s surface are extremely accurate, avoiding contributions due to the influence of underlying layers [17].

#### 2.3.2. Ultrastructure Analysis

For AFM data, the statistical roughness analysis was based on the height of each pixel in the image, as analyzed from the heightmap. The parameter *Rq* is the mean square roughness that is extremely sensitive to peaks and hollows, and is defined by Equation (1) [18]:

(1) $$\;\;\;\;R_{q}=\sqrt{\frac{1}{N}\Sigma_{i=1}^{N}z_{i}^{2\;}}$$

where *z* is each pixel height and *N* is the total number of pixels (in our case, 65,536), with the scan size standardized to 5 × 5 µm. Before the roughness analysis, maps were pretreated with a third-order polynomial fit [19], allowing more expressive height differences, especially promoted by sample preparation, to be minimized, resulting in a topography with more significant contributions from membrane structures or collagen fibers, depending on the analyzed layer.

##### Diameter Analysis

To analyze the diameters of collagen fibers and macromolecules in the epithelial basement membrane, Bowman’s layer, and stroma, approximately 150 (80) collagen fibers (macromolecules) from each layer were measured using the Nanoscope Analysis 2.0 software with the Section Analysis tool, taking the width distance at half height of each fiber. Values were expressed as mean ± SD

#### 2.3.3. Nanomechanical Analysis

**Figure 1:**
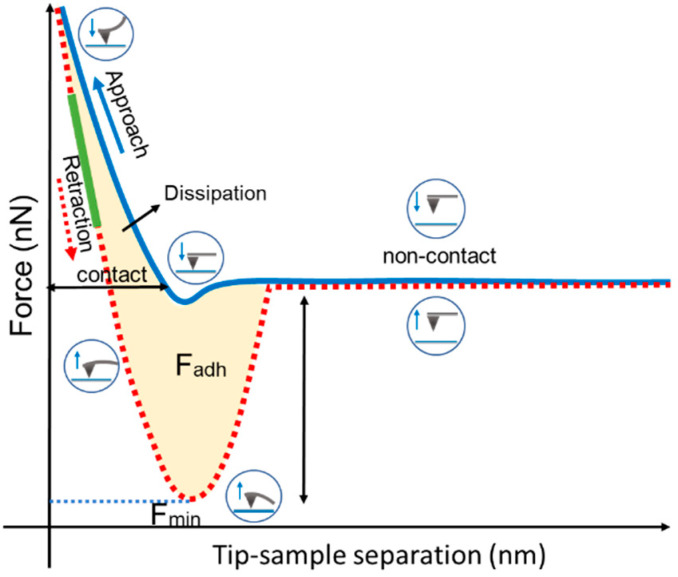
Schematic diagram of a typical force-versus-separation curve obtained with AFM. The blue line represents the approach cycle, and the dashed red curve represents the tip retraction curve. The yellow region represents the energy dissipation suffered by the cantilever during this cycle. Below the zero-force line, this dissipation corresponds to the adhesion force, which has its maximum value where the deflection, and consequently the force, is minimum (F_min_). The green line corresponds to the region from which the sample hardness was calculated. The diagrams in the circles represent the behavior of the cantilever during each step of the force-curve cycle.

The adhesion and stiffness of different cornea layers were calculated from all force curves. The adhesion force between the probe and the sample surface was obtained from each retraction curve (Figure 1, dashed curve in red), considered at the minimum cantilever deflection value (F_min_). This value represents the AFM probe’s resistance to leaving the sample surface.

For calculating the corneal layers’ stiffness, the slope values of the retraction curves were considered in intervals of 30–70% (Figure 1, green line). Surface stiffness is a parameter defined by:

(2) $$S=\frac{dF}{dz},$$

where *dF/dz* (N/m) is the derivative of the force applied by the cantilever on the surface of the sample in relation to the distance traveled by the probe during the cycle of retraction of the probe from the sample [20,21]. The indentations performed were limited in the equipment control software to 20 nm to ensure that the stiffness obtained corresponded to the analyzed layer. The PeakForce Quantitative NanoMechanics (QNM) operating mode is an extension of the intermittent contact mode (tapping mode) that allows the acquisition of force curves in each pixel of the image obtained at high frequencies (0.5–2 kHz), allowing the quantitative measurement of the sample properties at the nanoscale, generating high-resolution maps (with resolutions comparable to those of topographic images). As the QMN mode controls the force applied to the sample by the tip, the indentations on the sample’s surface are extremely accurate, avoiding contributions due to the influence of underlying layers [17].

#### 2.3.4. Statistical Analysis

Normal distribution following a single criterion was assessed using ANOVA and Tukey’s post hoc test, considering that the values were statistically significant when *p* < 0.05. Statistical analyses and graphs were created using the ORIGIN software. For nanomechanical data (adhesion and stiffness), one adhesion (stiffness) value per force curve was obtained, with 65536 force curves per map, and three maps per subject, for a total of 6 individuals (n = 6). For roughness data, a single *R_q_* value was acquired per map according to Equation (1), considering each height image pixel (256 × 256 pixels) for three maps for each sample (n = 6). For all data, the calculated error was the standard deviation (mean ± SD).

## 3. Results

### 3.1. Epithelial Layer

**Figure 2:**
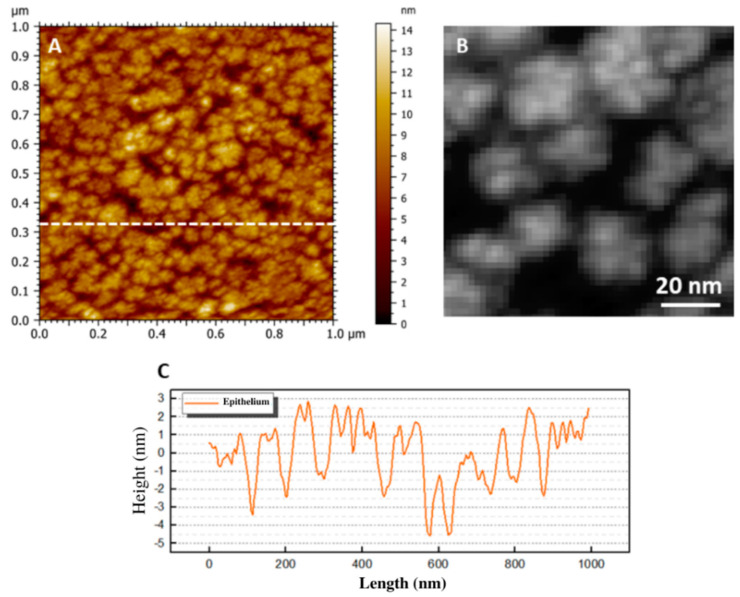
Topographic maps of the epithelial surface obtained using AFM. The 1 × 1 μm 2D images (**A**) show “microvilli”-like structures, with the epithelial cell membrane revealing various nanometer structures (**B**), indicating that these villi reached nanometer scales. A cross-section (**C**) in (**A**) shows nanovilli expressing the profile of peaks and valleys, with a diameter at the upper end of 9.8 ± 1.3 nm (in individual structures).

AFM images of the epithelial layer showed structures with projections from the cells that were very similar to microvilli (Figure 2A). The side scale bar is given in nanometers, and dark regions are seen as valleys or pores, while light regions are taller structures in the image. Despite the name “microvilli”, the ultrastructure was discovered to be much more complex (Figure 2B). The epithelial ultrastructure comprised several nanometric structures with a spicular arrangement. This magnification made it possible to observe nanometric structures with an average height of 23 ± 3.8 nm. The mean roughness was 4.69 ± 0.74 nm. This result suggested that the villi pattern reached nanometric scales (Figure 2C) with a nanovilli profile.

#### Epithelial Basement Membrane

**Figure 3:**
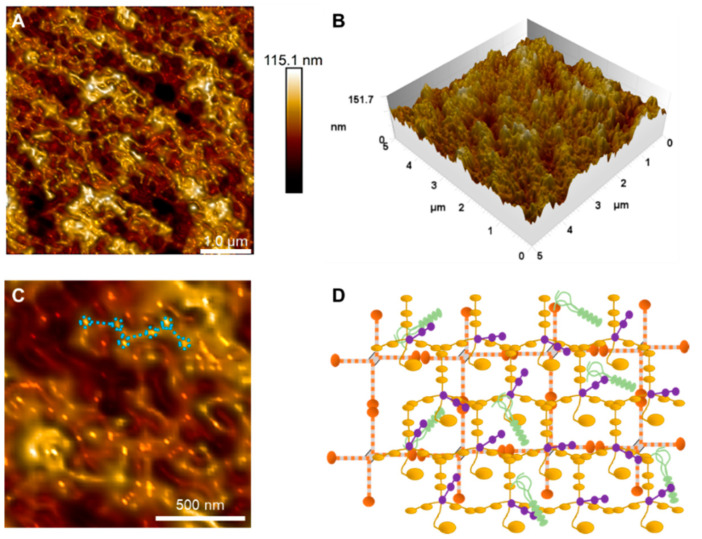
Topographic map of the basement membrane surface obtained using AFM. (**A**) Two-dimensional map of the scanning area of 5 × 5 μm and (**B**) its three-dimensional visualization. (**C**) Magnification shows the globular and linear ultrastructures on the basement membrane’s surface, indicated by blue dotted lines. (**D**) Schematic representation of the basement membrane in which the general molecular matrix led to this layer’s texture due to interactions between the main individual components.

In the images shown in Figure 3A,B, it is possible to observe globular structures connected by linear ones. Figure 3C shows a magnification of the structures with globular behavior (mean diameter 100.4 ± 4.6 nm) and linear parts (mean diameter 84.1 ± 2.7 nm), highlighted by the dashed structures in blue. The mean roughness was 13.40 ± 2.02 nm. To clarify, a schematic representation is shown (Figure 3D) of the basement membrane in which the general molecular matrix led to the texture of this layer due to interactions between the main individual components (collagen types IV and VI [22]; and laminins [23] and nidogens [24]).

### 3.2. Bowman’s Layer

**Figure 4:**
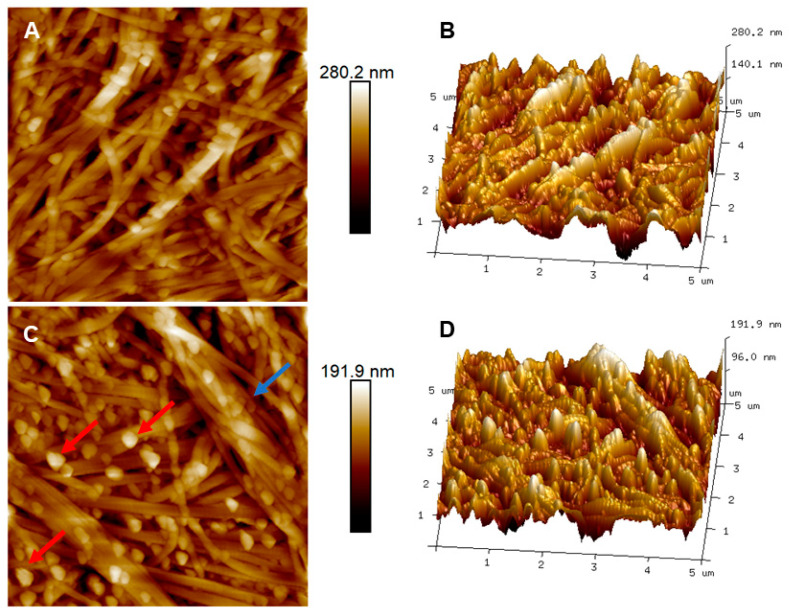
Two-dimensional and three-dimensional topographic maps of AFM from different regions of Bowman’s layer in human corneas. (**A**) Image of the 2D peripheral region and (**B**) its respective 3D view. (**C**) A 2D image of the central region and (**D**) its respective 3D image. Scan area = 5 × 5 μm.

The results of the analysis of the Bowman’s layers are presented in Figure 4, showing different portions (central and peripheral). It is possible to see the presence of collagen fibers arranged randomly, with a mean individual diameter of 134.7 ± 3.15 nm. The mean roughness was 36.30 ± 3.14 nm.

**Figure 5:**
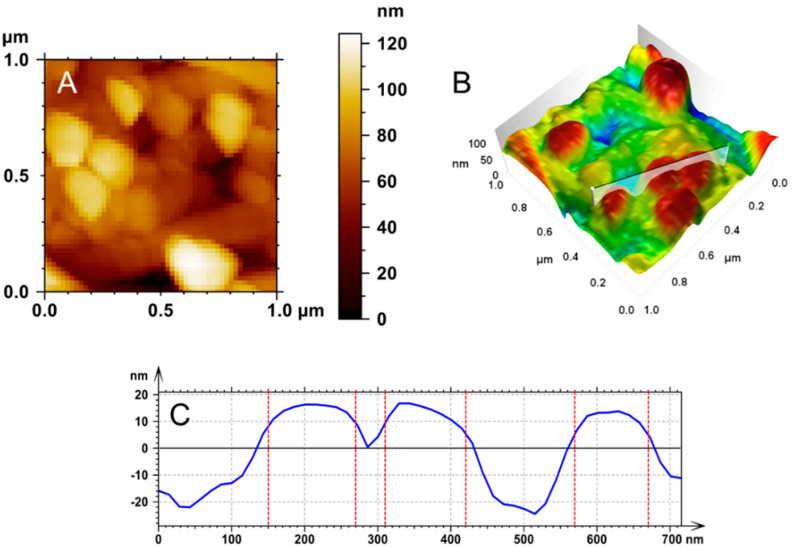
Two-dimensional (**A**) and three-dimensional (**B**) AFM topographic maps of the region of the Bowman’s layer presenting globular structures. The square scan area for all images is 1 × 1 μm. (**C**) A cross-section of the region marked in the image (**B**) shows the profile of three globules with diameters of 120, 110, and 100 nm, taken from the width at half height.

A detailed analysis of the collagen fibers is described in Figure 4C,D, in which it is possible to observe a random organization of collagen fibrils: bundles and twisting along the bundle (blue arrow). The individual fibrils for these maps had a mean diameter of 126.2 ± 6.2 nm. The larger bundle had a diameter ranging from 210 nm to 420 nm. In addition, spherical particles (red arrows) were detected with a medium diameter of 132.7 ± 5.1 nm. The entrance of macromolecules during the cornea scraping can explain this [25]. Figure 5 shows an image with details of the spherical structures present among the fibers.

### 3.3. Stroma

**Figure 6:**
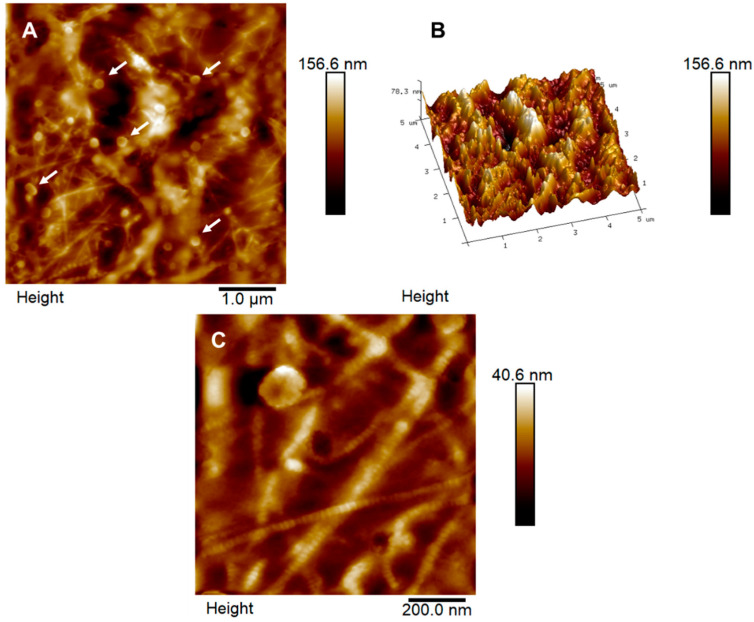
The surface of the human stroma (**A**) and its respective three-dimensional map (**B**) show the globular structures (white arrows) present in the topographic map in a 5 × 5 μm area. For more details on the stromal morphology, a topographic map with a 1 × 1 μm scan (**C**) shows the D-periodicity of collagen fibers.

Figure 6A,B show 2D and 3D topographic maps of the stroma. It is possible to observe the collagen fibers that made up this layer, which had an average diameter of 24.77 ± 2.77 nm and globular structures (white arrows) with a diameter of 156.91 ± 21.06 nm present after the process of scraping [25]. Its average roughness was 13.84 ± 3.88 nm. In Figure 6C, it is possible to see the collagen fibers in more detail and note the fiber’s D-periodicity.

### 3.4. Endothelium

**Figure 7:**
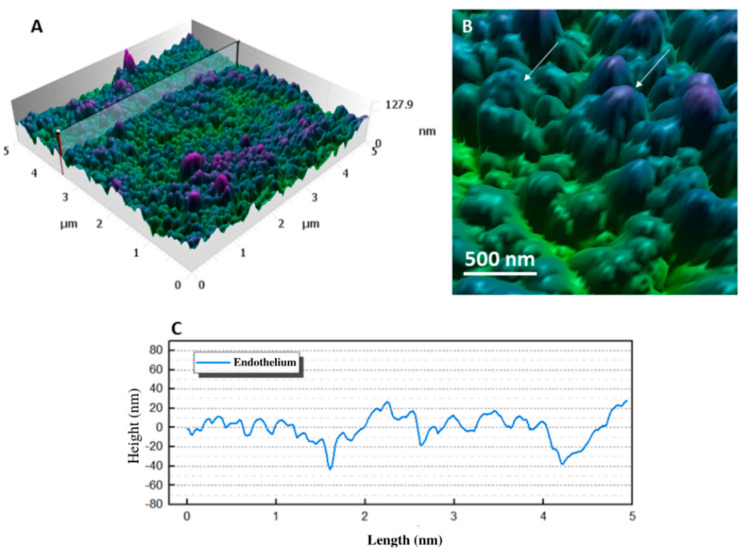
The surface of the endothelial layer. (**A**) Three-dimensional visualization of the 5 × 5 μm human corneal endothelial cell surface topographic map. (**B**) Enlarged image of this region showing the morphology of the ultrastructural structures. White arrows indicate globular structures compatible with macromolecules embedded in the membrane. (**C**) The heightmap cross-sections corresponding to the region marked in image (**A**).

Figure 7A shows a three-dimensional image of the endothelial cell membrane, with a maximum difference in the *z*-axis of 127.9 nm and showing several globular structures nanometric of 101 ± 2.2 nm diameter. Figure 7B shows an enlargement of the cell surface structures. Unlike those found in the basement membrane epithelial, these structures were not interconnected by linear networks, reaching 130 nm in maximum height. A striking feature of endothelial cells was the presence of a large population of vesicles in the plasma membrane that were 74 ± 3.5 nm in diameter, and were present on both cell surfaces [26]. The mean roughness was 12.70 ± 1.03 nm.

White arrows point to systems that appeared to “emerge” from the cell membrane, consistent with the macromolecules embedded in the cell membrane reported by Waring and colleagues [27].

### 3.5. Biophysical Properties of Layers

**Figure 8:**
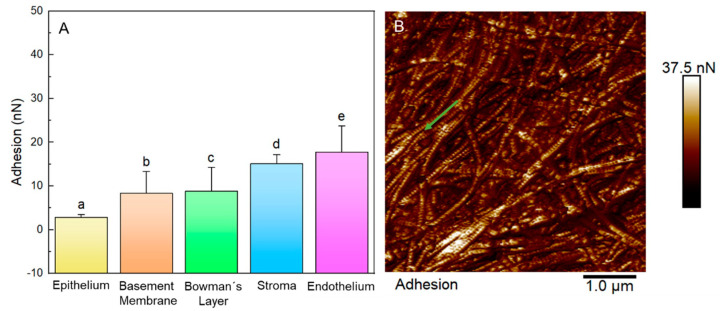
Adhesion data. (**A**) Adhesion force data for each layer. The letters on the graphs indicate the groups with significant differences in the ANOVA test with Tukey’s post hoc for *p* < 0.05. (**B**) A representative adhesion force map (5 × 5 µm) was created for the Bowman’s layer. The lighter areas of the map correspond to the highest values of adhesion forces. The green arrow shows the pattern of adhesion forces on collagen fibers.

**Figure 9:**
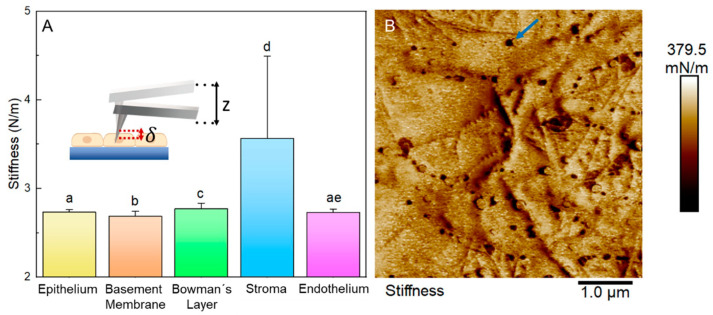
Stiffness values. (**A**) Graphs of local membrane stiffness values from different corneal layers. The inset picture represents the local layer deformation suffered by AFM probe force application. The letters on the graphs indicate the groups with significant differences in the ANOVA test with Tukey’s post hoc for *p* < 0.05. (**B**) A stiffness map was obtained from a 5 × 5 µm region of the stroma. The blue arrow emphasizes the local difference in stiffness due to macromolecules.

The adhesion as the stiffness of all the layers studied is presented in Figure 8 and Figure 9. The mean ± SD adhesion forces were: epithelium: 2.84 ± 0.63 nN; base membrane: 8.34 ± 4.98 nN; Bowman’s layer: 8.86 ± 5.37 nN; stroma: 15.06 ± 2.07 nN; and endothelium: 17.7 ± 6.03 nN (Figure 8A). A representative adhesion map of the Bowman’s layer is shown in Figure 8B. The green arrow highlights the difference in the adhesion forces of the fibers and their D-periodicity.

The mean ± SD local stiffness of each layer were: epithelium 2.73 ± 0.03 N/m; basal membrane, 2.69 ± 0.05 N/m; Bowman’s Layer, 2.77 ± 0.06 N/m; stroma: 3.56 ± 0.92 N/m; and endothelium, 2.73 ± 0.04 N/m (Figure 9A). Figure 9B shows a stiffness map obtained from a 5 × 5 µm region of the stroma. In this map, it is possible to observe how the presence of macromolecules in the stroma layer locally changed the stiffness of the layer (darker regions of the map).

## 4. Discussion

AFM images revealed the differences between the ultrastructures and biophysical properties of the different layers of the human corneas. However, this study could not detect a distinct pre-Descemet’s layer (DL). This could be partly due to the different preparation methodologies [7]. In the epithelial layer, the villi’s presence comes from these cells’ functional characteristics. Their function is to retain fluids in an eventual passage over its surface, preventing desiccation and cell death [28]. The results here suggested that this characteristic goes beyond the micrometric scale, with the presence of nanovilli observed with the AFM technique (Figure 2).

The lowest *R_q_* values were obtained for the epithelial layer. It should be noted that a 5-micrometer scan revealed a small region on the membrane of an epithelial cell that was about 20–30 μm in diameter. Thus, the ultrastructures observed in these maps were components of the cell membrane (proteins, lipids, and glycoproteins) and their arrangements, whose dimensions were at the nanometer or sub-nanometer scale. The high roughness values for the Bowman’s layer were associated with the arrangement of collagen fibers. The Bowman’s layer was about 8–15 μm thick and was characterized by the random organization of these fibers, which were often organized in bundles with diameters greater than 100 nm. This organization promoted a surface roughness superior to that of the other layers. Despite the diverse nature of the endothelial layer and basement membrane, the mean *R_q_* values were similar. This fact may be associated with both permeabilities, containing many pores in their ultrastructures.

The epithelial basement membrane is an extracellular matrix with a complex ultrastructure due to its different components, such as collagen types VI and IV, laminins, nidogens, and proteoglycans [29]. This extracellular matrix maintains the stability of epithelial cells, being a means of support for cell migration and the supply of nutrients and elimination of waste [30]. This layer is a major reservoir of growth factors and enzymes, and controls biological processes such as orderly cell migration and adhesion, wound healing, and tissue regeneration [15]. Without the correct combination of laminin isoforms, the interaction with other collagen molecules and proteoglycans is impaired, altering the morphology of the basement membrane and, consequently, rendering it dysfunctional [31,32].

The Bowman’s layer has an acellular structure composed of proteoglycans and collagen types I, III, V, and VI, which form collagen fibrils with diameters between 20–30 nm [33]. Collagen fibrils are randomly intertwined, creating a dense network [34]. The assembly of collagen fibrils takes place via connections of molecular structures. Collagen molecules are packaged to generate a pattern of repeated bands, known as D-periodicity, whose value depends on each tissue [35].

The stroma occupies 80–90% of the cornea, giving it mechanical strength and a characteristic shape. The stromal layer contains keratocytes, fibroblasts, and fibrocytes [13,32], presenting a selective extracellular matrix composed of macromolecules, such as proteoglycans and glycoproteins, and a dense network of type I collagen fibers with a diameter of 24.9–28 nm [36]. Its extracellular matrix produces sentinel cells—activated keratocytes—that serve as the first line of defense against cellular damage. Fibrocytes produce collagen fibers after fibroblasts mature, and keratocytes begin in a continuous network that functions as a gap junction [36].

The endothelium is formed by a single layer of hexagonal cells forming a mosaic covering the cornea’s posterior region. Adjacent endothelial cells share extensive lateral interdigitations, and the lateral portion of the endothelial cell membrane contains a high density of sodium (Na^+^) and potassium (K^+^) pumps. Endothelial cells regulate the amount of fluid in the stromal layer, drawing fluid from this layer into the aqueous humor [27]. The plasma membrane of the endothelial cell has numerous pinocytotic vesicles on its anterior and posterior surface that perform transport and secretion functions. The membrane also contains embedded protein macromolecules such as “icebergs” that are free to move laterally within the membrane [27]. These proteins may include drug or hormone receptors, antigenic sites, and enzymes for active transport [27]. It was possible to observe the ultrastructures present in the endothelial cell membrane using the AFM technique, as shown in Figure 7.

In cells, stiffness is mainly governed by the architecture of the cytoskeleton and the composition and distribution of membrane components. The cytoskeleton’s complex filament architecture is composed of actin filaments, intermediate filaments, and microtubules [37]. Individually, these components are extremely rigid. However, when structured in a network of reticulated filaments, they result in a malleable material with dynamic properties different from the individual properties of each component [37]. Epithelial cells form a tissue whose function in the cornea is protection against pathogen retention of the tear film; these cells are the first barrier of the eye in the external surface.

The stroma and Bowman’s layer showed the highest mean stiffness values due to the dense intertwining between collagen fibrils. This result supported the hypothesis that the stroma provides stability to the shape and structure of the cornea due to its rigidity [11,32]. The high dispersions of stroma stiffness values demonstrated the influence of the heterogeneity of collagen fibers and other structures on the surface of this layer. Topographic maps supported this result; in the stromal height images, the visualization of fiber distribution decreased due to increased heterogeneity of components, such as macromolecules and structures that suggested gap junctions of keratocytes cultured in collagen gels [36]. The dispersion of stiffness values for the basement membrane was similar to the values for the Bowman’s layer, probably due to the presence of collagen components. Although both layers have collagen as their main component, the type of collagen and the arrangement formed by the interaction with other components confer different mechanical properties on each layer. Changes in basement membrane rigidity can impair the processes of migration, adhesion, and proliferation of epithelial cells [38].

On the other hand, corneal endothelial cells need to withstand hydrodynamic pressures from the aqueous humor. The endothelial layer functions as a semi-permeable barrier, controlling the exchange of fluids, electrolytes, and proteins between the aqueous humor and the cornea. The integrity of this layer is crucial to maintaining corneal deturgescence [39]. Corneal epithelial and endothelial cells have major cytoskeleton components: actin filaments, intermediate filaments, and microtubules [40].

Regarding the adhesion results, it is important to address that the adhesion maps did not have the same physical nature as the height maps. During QNM measurements, the maps were obtained from the force curves composed of cycles of approximation and retraction of the tip relative to the sample [17]. The adhesion forces combine electrostatic, van der Waals, capillary, and forces promoted by chemical bond breakage [21,41,42]. Especially with non-functionalized probes (such as in this research), the adhesion forces are taken as non-specific interactions, and it is impossible to separate the contribution of each of these forces. However, the probes used here to analyze all samples were made of the same material and had the same specifications (geometry, tip radius, etc.), just as the experiments for all layers were performed under the same conditions (temperature and humidity of the air). Differences in the contrast of adhesion forces were mainly related to van der Waals and electrostatic interactions, meaning different components of the particle surface [16,20].

Butt et al. [41] pointed out that, especially in measurements in air, contributions of electrostatic forces are expected, particularly in non-conductive samples and in low air humidity, when charge dissipation is ineffective. Changes arising from electrostatic forces result in a greater or lesser accumulation of charge on the surface, which is again associated with the layer composition and/or structure. Because the interactions between the probe and sample occurred in minimum humidity conditions, 42%, there was a low influence of meniscus forces on adhesive forces [41]. Roughness data reinforced the hypothesis that a high roughness retains a moisture layer, where the basement membrane, stroma, and endothelium had values very close to roughness, but far from adhesion. The charge dissipation was ineffective for biological materials analyzed in the air and under low humidity conditions, corroborating the greater influence of electrostatic and van der Waals forces.

Due to the characteristic of containing numerous ionic pumps, the endothelium has a higher value of adhesive forces. The endothelial layer handles the cornea’s transparency and maintains the stroma’s dehydration, creating a leaky barrier between the aqueous humor and the stroma [43]. Thus, stromas containing proteoglycans with a high negative content, expressions of cytokines, and adhesion molecules [36] can select their extracellular matrix by recruiting and activating cells, such as hydrophilic keratan sulfate, making the stroma progressively dehydrated and compact [44]. With this ability to select molecules and ionic components, the stroma had the second-highest adhesion value.

For the Bowman’s layer, there was evidence that its physiological characteristics originated from a pyramidal stroma. The Bowman’s layer is a slight modification of the anterior stroma due to the presence of the keratocan protein [44]. Therefore, by modifying a thin part of the primary anterior stroma, making it acellular, the Bowman’s layer matures and develops [44], losing part of the extracellular matrix and decreasing the adhesion values. The basement membrane plays a key role in modulating cellular components such as proliferation, differentiation, and migration [45]. Through regulating parts and as an epithelial extracellular matrix, the basement membrane has molecules capable of forming complexes with other continuums, facilitating the interaction between molecules of the basement membrane and cell surface in a more selective way, characterizing filtration [46]. The epithelium had the lowest value of adhesive forces due to its barrier function against the external environment. Its low adhesion value was due to its nanovilli, which increased the contact area of epithelial cells retaining the lacrimal fluid and the presence of proteins on its surfaces, such as IFN-λ interferon and the IFNλR1 receptor, which function as a defense system [47]. Thus, this layer must have good malleability to promote these processes. Determining these biomechanical markers is important to classify healthy tissues biophysically and for later comparison with those affected by pathologies.

## 5. Conclusions

This tool analyzed the ultrastructure and biomechanical properties of human corneal layers. High-resolution topographic maps revealed the different ultrastructures present in each layer of the cornea. The adhesion and local stiffness values, obtained through the analysis of the AFM force curves, also showed the differences between the corneal layers due to their specific components.

Through the analysis of the ultrastructure of the surface of epithelial cells of human corneas, it was possible to verify that the microvilli of this surface presented subunits of nanometric size, suggesting a fractal pattern of organization of the microvilli. For the surface of the basement membrane, it was possible to observe the organization of its molecular components, revealing structures suggested as collagen fibers and laminin molecules. This result underscored the surprising resolving power of the AFM. We observed anisotropy in the structuring of collagen fibrils for the Bowman’s layer, even on a sub-micrometer scale. The ultrastructures observed on the surface of endothelial cells revealed nanometric structures emerging from the cell membrane, compatible with the macromolecules embedded in the cell membrane. It is important to emphasize that the characterization with such resolution of many of these morphological findings is unprecedented in the literature.

Analyses of the biomechanical properties of the different layers of the human cornea have also been extremely promising. The epithelium, basement membrane, Bowman’s layer, and endothelium have distinct adhesion forces and stiffness properties. The data presented in this work add a great deal of knowledge regarding the characterization of human corneas’ physical properties and ultrastructures. Understanding how each layer of the cornea behaves at the nanometer scale is essential to understanding tissue physiology fully and how corneal diseases can affect such features.

## Data Availability

The datasets generated during and/or analysed during the current study are available from the corresponding author on reasonable request.

## References

[1] Lwigale P.Y. (2015). Corneal Development. Progress in Molecular Biology and Translational Science.

[2] Foster J.W., Wahlin K., Adams S.M., Birk D.E., Zack D.J., Chakravarti S. (2017). Cornea organoids from human induced pluripotent stem cells. Sci. Rep..

[3] Lombardo M., Lombardo G., Carbone G., Santo M.P.D., Barberi R., Serrao S. (2012). Biomechanics of the Anterior Human Corneal Tissue Investigated with Atomic Force Microscopy. Investig. Ophthalmol. Vis. Sci..

[4] DelMonte D.W., Kim T. (2011). Anatomy and physiology of the cornea. J. Cataract Refract. Surg..

[5] Lekhanont K., Panday V., Akpek E.K. (2009). Permanent keratoprostheses. Corneal Surgery.

[6] Taher E.E., Elalfy M., Elsawah K. (2020). Stem cell therapies in ocular repair, regeneration, and diseases. Mesenchymal Stem Cells in Human Health and Diseases.

[7] Dua H.S., Faraj L.A., Said D.G., Gray T., Lowe J. (2013). Human corneal anatomy redefined: A novel pre-descemet’s layer (Dua’s Layer). Ophthalmology.

[8] Martin R. (2018). Cornea, and anterior eye assessment with slit lamp biomicroscopy, specular microscopy, confocal microscopy, and ultrasound biomicroscopy. Indian J. Ophthalmol..

[9] Gamidov A.A., Baryshev K.V., Perevozchikov K.A., Surnina Z.V. (2020). Atomic force microscopy in the study of retinal structure. Vestn. Oftalmol..

[10] Alhasawi A. (2016). Microstructural Imaging of the Eye and Mechanical Mapping of Retinal Tissue using Atomic Force Microscopy (AFM). Doctoral Dissertation.

[11] Grant C.A., Thomson N.H., Savage M.D., Woon H.W., Greig D. (2011). Surface characterisation and biomechanical analysis of the sclera by atomic force microscopy. J. Mech. Behav. Biomed. Mater..

[12] Diakonis V.F., Likht N.Y., Yesilirmak N., Delgado D., Karatapanis A.E., Yesilirmak Y., Fraker C., Yoo S.H., Ziebarth N.M. (2016). Corneal elasticity after oxygen enriched high intensity corneal cross linking assessed using atomic force microscopy. Exp. Eye Res..

[13] Dias J., Diakonis V.F., Lorenzo M., Gonzalez F., Porras K., Douglas S., Avila M., Yoo S.H., Ziebarth N.M. (2015). Corneal stromal elasticity and viscoelasticity assessed by atomic force microscopy after different cross linking protocols. Exp. Eye Res..

[14] Seifert J., Hammer C.M., Rheinlaender J., Sel S., Scholz M., Paulsen F., Schäffer T.E. (2014). Distribution of Young’s modulus in porcine corneas after riboflavin/UVA-induced collagen cross-linking as measured by atomic force microscopy. PLoS ONE.

[15] Abrams G.A., Schaus S.S., Goodman S.L., Nealey P.F., Murphy C.J. (2000). Nanoscale Topography of the Corneal Epithelial Basement Membrane and Descemet’s Membrane of the Human. Cornea.

[16] Belikov S., Alexander J., Wall C., Yermolenko I., Magonov S., Malovichko I. Thermal tune method for AFM oscillatory resonant imaging in air and liquid. Proceedings of the 2014 American Control Conference.

[17] Adamcik J., Berquand A., Mezzenga R. (2011). Single-step direct measurement of amyloid fibrils stiffness by peak force quantitative nanomechanical atomic force microscopy. Appl. Phys. Lett..

[18] Dufrêne Y.F. (2009). Atomic force microscopy: A powerful molecular toolkit in nanoproteomics. Proteomics.

[19] Gong Y., Misture S.T., Gao P., Mellott N.P. (2016). Surface Roughness Measurements Using Power Spectrum Density Analysis with Enhanced Spatial Correlation Length. J. Phys. Chem. C.

[20] Costa L.M., Silva C.R., Soares A.M., Menezes A.S., Silva M.R., Amarante A.F., Costa E.F., Alencar L.M. (2020). Assessment of biophysical properties of Haemonchus contortus from different life cycle stages with atomic force microscopy. Ultramicroscopy.

[21] Amorim M.D.S.D.N., Batista J.A., Maia F., Fontes A., Santos-Oliveira R., Rebelo Alencar L.M. (2021). Alencar. New Insights into Hemolytic Anemias: Ultrastructural and Nanomechanical Investigation of Red Blood Cells Showed Early Morphological Changes. SSRN J..

[22] Furthmayr H., Wiedemann H., Timpl R., Odermatt E., Engel J. (1983). Electron-microscopical approach to a structural model of intima collagen. Biochem. J..

[23] Mak K.M., Png C.Y.M. (2015). Type VI Collagen: Biological Functions and Its Neo-epitope as Hepatic Fibrosis Biomarker. Biomarkers in Liver Disease.

[24] Fox J.W., Mayer U., Nischt R., Aumailley M., Reinhardt D., Wiedemann H., Mann K., Timpl R., Krieg T., Engel J. (1991). Recombinant nidogen consists of three globular domains and mediates binding of laminin to collagen type IV. EMBO J..

[25] Torricelli A.A.M., Marino G.K., Santhanam A., Wu J., Singh A., Wilson S.E. (2015). Epithelial basement membrane proteins perlecan and nidogen-2 are up-regulated in stromal cells after epithelial injury in human corneas. Exp. Eye Res..

[26] Predescu D., Palade G.E. (1993). Plasmalemmal vesicles represent the large pore system of continuous microvascular endothelium. Am. J. Physiol.-Heart Circ. Physiol..

[27] Waring G.O., Bourne W.M., Edelhauser H.F., Kenyon K.R. (1982). The corneal endothelium. Normal and pathologic structure and function. Ophthalmology.

[28] Tatiana M. (2016). Atlas Digital de Biologia Celular e Tecidual.

[29] Byström B., Virtanen I., Rousselle P., Miyazaki K., Lindén C., Domellö F.P. (2007). Laminins in normal, keratoconus, bullous keratopathy and scarred human corneas. Histochem. Cell Biol..

[30] Torricelli A.A.M., Singh V., Santhiago M.R., Wilson S.E. (2013). The Corneal Epithelial Basement Membrane: Structure, Function, and Disease. Invest. Ophthalmol. Vis. Sci..

[31] Stanley R. (2016). BSAVA manual of canine and feline ophthalmology. Aust. Vet. J..

[32] Gozzo F. (2009). Caracterização Morfológica de Tecidos Oculares por Microscopia de Força Atômica. Master’s Dissertation.

[33] Eghrari A.O., Riazuddin S.A., Gottsch J.D. (2015). Overview of the Cornea. Progress in Molecular Biology and Translational Science.

[34] Chen Z., You J., Liu X., Cooper S., Hodge C., Sutton G., Crook J.M., Wallace G.G. (2018). Biomaterials for corneal bioengineering. Biomed. Mater..

[35] Stylianou A. (2017). Atomic Force Microscopy for Collagen-Based Nanobiomaterials. Nanomaterials.

[36] Fukuda K. (2020). Corneal fibroblasts: Function and markers. Exp. Eye Res..

[37] Rebêlo L.M., de Sousa J.S., Filho J.M., Schäpe J., Doschke H., Radmacher M. (2013). Microrheology of cells with magnetic force modulation atomic force microscopy. Soft Matter.

[38] Molladavoodi S., Kwon H.-J., Medley J., Gorbet M. (2015). Human corneal epithelial cell response to substrate stiffness. Acta Biomater..

[39] Yuan S. (2009). Endothelial contractile cytoskeleton and microvascular permeability. CHC.

[40] Last J.A., Russell P., Nealey P.F., Murphy C.J. (2010). The Applications of Atomic Force Microscopy to Vision Science. Invest. Ophthalmol. Vis. Sci..

[41] Butt H.-J., Cappella B., Kappl M. (2005). Force measurements with the atomic force microscope: Technique, interpretation and applications. Surf. Sci. Rep..

[42] Cardoso-Lima R., Souza P.F.N., Guedes M.I.F., Santos-Oliveira R., Alencar L.M.R. (2021). SARS-CoV-2 Unrevealed: Ultrastructural and Nanomechanical Analysis. Langmuir.

[43] Zhu C., Joyce N.C. (2004). Proliferative Response of Corneal Endothelial Cells from Young and Older Donors. Invest. Ophthalmol. Vis. Sci..

[44] Quantock A.J., Young R.D. (2008). Development of the corneal stroma, and the collagen-proteoglycan associations that help define its structure and function. Dev. Dyn..

[45] Last J.A., Liliensiek S.J., Nealey P.F., Murphy C.J. (2009). Determining the mechanical properties of human corneal basement membranes with atomic force microscopy. J. Struct. Biol..

[46] de Souza R.S., Pinhal M.A.D.S. (2011). Interações em processos fisiológicos: A importância da dinâmica entre matriz extracelular e proteoglicanos. Arq. Bras. Ciên. Saúde.

[47] Miner J.J., Platt D.J., Ghaznavi C.M., Chandra P., Santeford A., Menos A.M., Dong Z., Wang E.R., Qian W., Karozichian E.S. (2020). HSV-1 and Zika Virus but Not SARS-CoV-2 Replicate in the Human Cornea and Are Restricted by Corneal Type III Interferon. Cell Rep..

